# The Fungoid Theory of Cholera

**Published:** 1871-06

**Authors:** 


### The Fungoid Theory of Cholera.

Mr. T. R. Lewis, M. D., who was specially appointed to investigate the theories of Hallier and Petenkofer with regard to cholera, announces, after long and careful examination, that.

1. No "cysts" exist in choleraic discharges which are not found under other conditions. 2. Cysts or "sporangia" of fungi are but very rarely found under any circumstances in alvine discharges. 3. No Special fungus has been developed in cholera stools, the fungus described by Hallier being certainly not confined to such stools. 4. The still and active conditions of the observed animalculæ are not peculiar to this disease, but may be developed in nitrogenous material even outside the body. 5. The flakes and corpuscles in rice-water stools do not consist of epithelium, nor of its *debris;* but their formation appears to depend upon the effusion of blood plasma; and the "peculiar bodies" Parkes found therewith correspond very closely in their microscopic and chemical characters, as well as in their manifestations of vitality, to the corpuscles which are known to form in such fluid; these are generally, to a greater or less degree, associated with blood-cells, even when the presence of such if not suspected, especially as the disease tends toward a fatal termination, when the latter have frequently been seen to replace the former altogether. 6. No sufficient evidence exists for considering that vibrones, and such-like organisms prevail to a greater extentin the discharges from persons affected with cholera, than in the discharges of other persons, diseased or healthy; but that the vibriones, bacteria, and monads (micrococcus) may not be *peculiar in their nature*, for these *do* vary, and may not be the product of a peculiar combination of circumstances, and able to give origin to peculiar phenomena in a predisposed person, is "not proven."—*Med. and Surg. Reporter.*

